# Targeted High-Throughput Sequencing Identifies Predominantly Fungal Pathogens in Patients with Clinically Infectious, Culture-Negative Endophthalmitis in South India

**DOI:** 10.3390/microorganisms7100411

**Published:** 2019-10-01

**Authors:** Jaishree Gandhi, Rajagopalaboopathi Jayasudha, Poonam Naik, Savitri Sharma, Vivek Pravin Dave, Joveeta Joseph

**Affiliations:** 1 Jhaveri Microbiology Centre, Prof. Brien Holden Eye Research Centre, L. V. Prasad Eye Institute, Kallam Anji Reddy campus, Hyderabad, Telangana 500034, India; jaishreegandhi123@gmail.com (J.G.); jayasudha001@gmail.com (R.J.); naikpoonam92@gmail.com (P.N.); savitri@lvpei.org (S.S.); 2 Smt. Kannuri Santhamma Centre for vitreoretinal diseases, L. V. Prasad Eye Institute, Kallam Anji Reddy campus, Hyderabad, Telangana 500034, India; vivekoperates@yahoo.co.in

**Keywords:** endophthalmitis, high throughput sequencing, culture-negative

## Abstract

To evaluate the clinical utility of high-throughput sequencing (HTS) approach-based analysis of the bacterial and fungal genome in vitreous fluids from patients clinically diagnosed as endophthalmitis, we subjected 75 vitreous fluids from clinically presumed infectious endophthalmitis patients to high-throughput sequencing (Illumina HiSeq 2500) after DNA extraction and amplification of the 16S rRNA for the detection of bacteria, and ITS 2 region for detection of fungal pathogens. As controls, we included vitreous biopsies from 70 patients diagnosed with other non-infectious retinal disorders. Following the construction of the curated microbial genome database and filtering steps to reduce ambiguousness/contaminants from the environment, the paired reads were analyzed. Our HTS reads revealed in almost all cases the same organism that was grown in culture (bacterial-14/15, fungal 3/3) by conventional microbiological workup. HTS additionally diagnosed the presence of microbes in 42/57 (73.7%) patients who were conventionally negative (fungal pathogens in 36/57, bacterial pathogens in 11/57, including five cases that showed the presence of both bacterial and fungal organisms). *Aspergillus* sp., *Fusarium* sp., *Exserohilum* sp., and *Candida* sp. were the most predominant genera in our cohort of culture-negative endophthalmitis cases. Heat map based microbial clustering analysis revealed that these organisms were taxonomically similar to the species identified by conventional culture methods. Interestingly, 4/70 control samples also showed the presence of bacterial reads, although their clinical significance is uncertain. HTS is useful in detecting pathogens in endophthalmitis cases that elude conventional attempts at diagnosis and can provide actionable information relevant to management, especially where there is a high index of suspicion of fungal endophthalmitis, particularly in tropical countries. Outcome analyses and clinical trials addressing the success and cost savings of HTS for the diagnosis of endophthalmitis are recommended.

## 1. Introduction

Endophthalmitis is a purulent vision-threatening infection of the intraocular fluids and is reported to vary by geographic location in incidence and cause, following ocular surgery or injury and rarely by hematogenous spread [1]. The clinical outcome depends on the pathogenesis of the organism and clinical intervention. Identification and characterization of the causal pathogen from routine culture media is limited due to the early administration of broad-spectrum or prophylactic antimicrobial drugs, as well as organisms that are fastidious or slow-growing [2]. However, a common concern with conventional testing methods is the limitation in the breadth of pathogens detected, and clinicians are often left with negative results and the persisting question of whether the condition was actually caused by an infection or just an inflammatory reaction. While direct sequencing of the 16S rRNA/ITS region can be applied on clinical specimens to detect the presence of microorganisms [3,4], in low pathogen loads or polymicrobial infections it is usually challenging to sort out ambiguous signals from mixed chromatograms of samples, and sequences often remain unidentified/misidentified. High-throughput sequencing (HTS) also termed as next-generation sequencing (NGS), is a novel platform with the potential to simultaneously detect and independently sequence virtually all the DNA sequences of the infectious agents present in a sample [5,6,7]. However, this unbiased amplification results in a large number of reads of both host and pathogen DNA, and the complexities in detecting and interpreting millions of sequences to identify the microbe(s) of interest is considerable and challenging. Targeted HTS refers to the selective capture or amplification of specific genomic regions of interest prior to massive parallel sequencing, compared to unbiased random amplification which results in the sequencing of all the nucleic acids, including host and pathogen nucleic acids, and millions of reads need to be analyzed to identify the pathogen(s) of interest. Targeted HTS, on the other hand, provides better sensitivity and specificity along with ease of downstream analysis and a lower cost by allowing more samples to be tested in one run [8].

To cross the divide between the conventional microbiological methods and whole-genome sequencing, a culture free platform using targeted HTS would be an excellent, less complicated approach, with a lower cost to detect pathogens for implementation in diagnostic laboratories. We have previously shown that NGS is a good tool for microbial research in endophthalmitis [9], but the next big challenges will be the standardization and validation of procedures and bioinformatics pipeline that address the specific challenges for clinical standards, especially in culture-negative cases. HTS is more likely to become a future diagnostic tool in routine ocular microbiological laboratories. Therefore, in this study, we tested 75 vitreous fluids using the targeted HTS assay, to evaluate its feasibility in an ocular clinical setting for the diagnosis of culture-negative endophthalmitis. Finally, the scope and impact of this method to diagnose pathogens in vitreous biopsies were evaluated within a clinical context.

## 2. Results

The test group included seventy-five patients (54 males and 21 females with a mean age of 39 ± 24 years) who presented with characteristics of infectious endophthalmitis, and the source of infection in these test samples were found to be post-traumatic (52%, 39 patients), post-surgical (37.3%, 28 patients), and endogenous spread (10.6%, 8 patients). Whereas 70 vitreous control samples collected from 47 males and 23 females with a mean age of 45 ± 18 were included in the study. The details are described in Table 1.

### 2.1. Microbiology Culture

From 75 test samples, 18 were culture-positive (Table 2 and Table 3), of which 15 grew bacteria and three showed the presence of fungus. Among the most common gram-positive bacteria, ten samples were culture-positive for *Streptococcus* sp. and *Staphylococcus* sp. while two samples showed the presence of *Enterococcus* sp. and two were positive for gram-positive bacilli and the remaining one sample was culture-positive for *Klebsiella pneumoniae*. In the case of fungus, two were culture-positive for *Candida* sp. and the remaining one grew *Fusarium solani*.

### 2.2. Taxonomic Analysis by NGS

**Figure 1:**
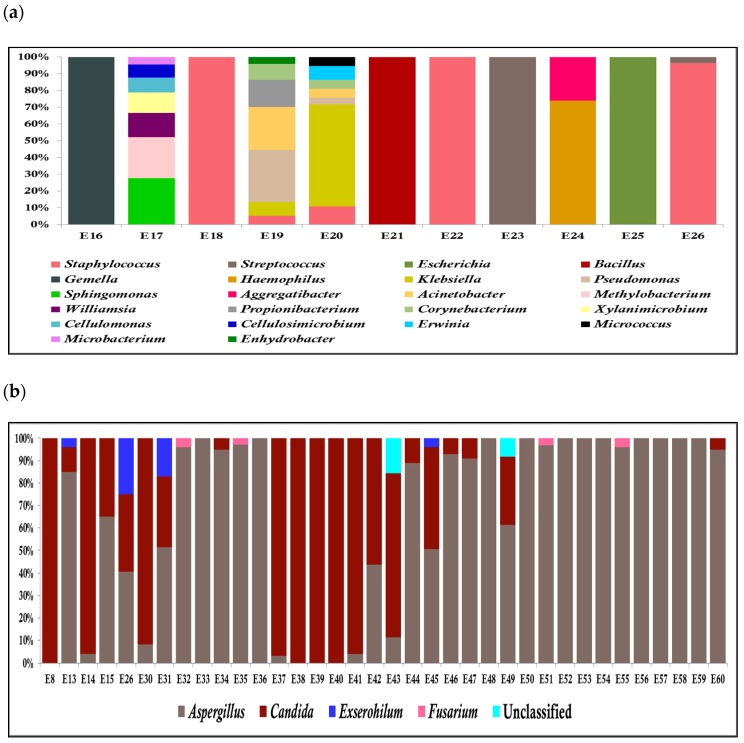
Stacked bar graph for culture-negative samples by HTS. (**a**) Stacked bar graph displaying the relative abundances of the bacterial genera obtained by HTS of culture-negative vitreous samples.

HTS of the V3-V4 region of 26 samples on the Illumina platform generated up to 16.1 million high-quality reads and average reads per sample were 0.61 million reads ± 0.37 million reads [s.d] and clustered into 1910 OTUs (664 reference OTUs and 1246 de novo OTUs). With the ITS2 region amplicon of 39 samples, up to 25.53 million reliable reads were generated with the mean of 0.65 ± 0.31 million reads per sample, which clustered into 394 OTUs (104 reference OTUs and 290 de novo OTUs). It is interesting to note that the number of fungal OTUs is significantly lower than the bacterial OTUs. This is presumably because of less diversity of fungal pathogens involved in endophthalmitis. An analysis of 75 patient samples with presumed infectious endophthalmitis by HTS, identified microbial existence in 60 patients, of which only 18 were conventional culture-positive for bacteria (15) and fungus (3), as shown in Table 2a,b. Among 57 culture-negative cases, 11 cases showed the presence of bacteria (Figure 1a), and 36 cases unveiled the presence of fungus (Figure 1b), in which 5 cases showed the presence of both bacteria and fungi. A total of 15 out of 75 samples were negative for both microbial cultivation as well as HTS.

### 2.3. Agreement between Culture and HTS Techniques for Culture-Positive Samples

HTS results for culture-positive cases showed a high-quality match in 14/15 bacterial cases and 3/3 fungal cases (Table 2 and Table 3), and the same pathogen was identified. In 1/15 cases, there was a complete mismatch of the bacteria as *Corneybacterium pseudodiphtherticum* grew in culture while HTS analysis showed the presence of *Pseudomonas* sp. In the case of fungus, predominantly *Candida* sp. and *Fusarium* sp. were identified by HTS (Table 3).

### 2.4. HTS Analysis for Culture-Negative Endophthalmitis

HTS detected clinically significant fungal pathogens in 36/57 samples and bacterial pathogens in 11/57, including five cases that showed the presence of both bacterial and fungal organisms of which are conventionally culture-negative. HTS analysis for bacterial culture-negative cases was predominantly positive for *Streptococcus* sp.*, Staphylococcus* sp., *Bacillus* sp.*, Gemella* sp., and *Klebsiella* sp. (Figure 1a), while in the case of culture-negative fungus, *Apergillus* sp.*, Candida* sp.*, Exserohilum* sp*.,* and *Fusarium* sp. (Figure 1b) were predominant. 

### 2.5. Heatmap with Bacterial and Fungal Genera

**Figure 2:**
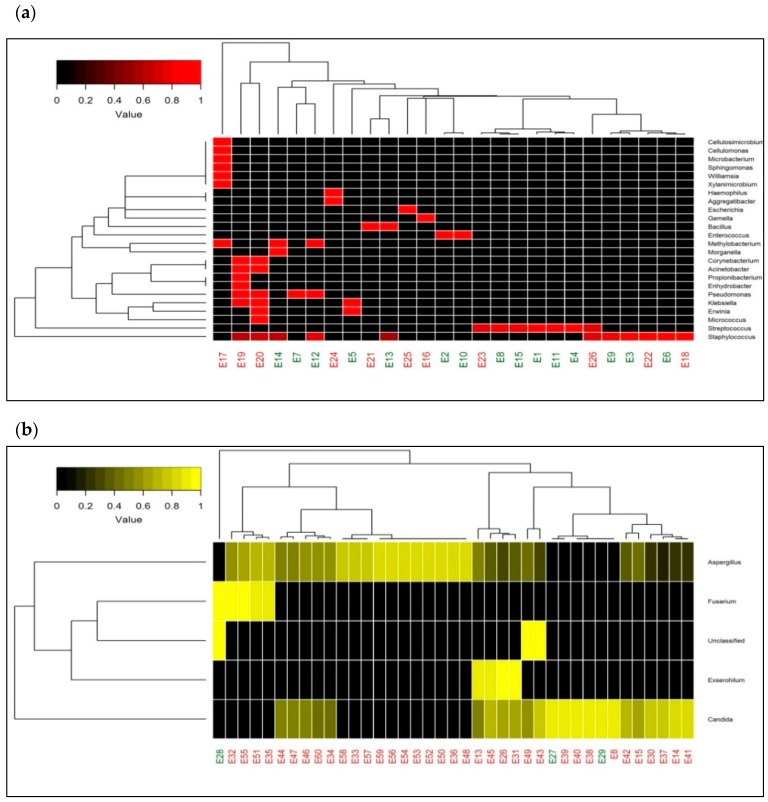
Taxonomic heatmap representing microbial abundance in patient samples. (**a**) Heatmap depicting bacterial diversity and its relative abundance from both culture-positive and culture-negative vitreous fluids of patients with endophthalmitis clinically. (**b**) Heatmap depicting fungal abundances in vitreous of patients with both culture-positive and culture-negative endophthalmitis. The color legend on the top left side indicates the average relative abundances of these genera in each sample.

A heatmap depicting the relative abundances of 24 bacterial genera in 26 samples in two-dimensional space, did not segregate culture-positive and culture-negative samples into two different clusters (Figure 2a). It reveals that the abundance of bacterial genera did not vary across the culture positive and negative samples. The heatmap with fungal genera of 39 samples also failed to segregate culture-positive and culture-negative samples (Figure 2b). It is also interesting to note that the samples were predominantly dominated by *Aspergillus* sp. and *Candida* sp. The hierarchical clustering in both heatmaps (Figure 2a,b) discloses that the causative organisms (bacteria/fungi) of endophthalmitis were similar in culture-positive and negative cases.

Interestingly four of the 70 control samples also showed the presence of bacterial pathogens by HTS, as shown in Table 4.

## 3. Discussion

Precise diagnosis and accurate treatment of endophthalmitis depends largely on microbiological evidence and goes a long way in preventing irreversible ocular damage. Unfortunately, the number of patients coming to our institute (being a tertiary referral center) have had previous administration of antibiotics and are often severe and difficult to diagnose, leading to an increase in culture-negative infections. We have previously reported that next-generation sequencing of vitreous samples could be used for the diagnosis of infectious endophthalmitis and concluded in our small size, that culture-negative cases are actually not devoid of microorganisms [9]. As an extension of our earlier study, we now included mainly patients with culture-negative infections, but presumed clinically as infectious endophthalmitis, in this study for validation and its diagnostic implications. It is in the interest of patients, that ocular microbiology laboratories should always be ready to adapt new technology that complements traditional workflow for better diagnostics and HTS of vitreous samples is a step in that direction, especially in countries like ours with a tropical climate, and the etiology of infections is different from that of the west. Currently, HTS of the whole genomic DNA suffers from two major limitations, one being the lack of a reference database/software that allows reliable species identification after filtering contaminant DNA, and the second major limitation of the method is the cumbersome filtering and bioinformatics analysis involved for each sample within a clinically relevant time frame necessary for timely treatment of infections and thereby antibiotic resistance control [10]. The proportion of human DNA that is sequenced can, however, be reduced by targeting amplicon sequencing using conserved primers, followed by library preparation [8]. One common variation of this technology is the PCR amplification of 16S rRNA targeting the hypervariable regions (V1–V9) in the case of bacteria and the ITS2 region in the case of fungi, followed by HTS of the resulting amplicon. This would not only result in decreasing the costs significantly but also help in reduced turnaround times for implementation in clinical diagnostics. 

The detection of organisms from the culturally negative vitreous of patients in this study is in the presence of clinical suspicion of infection and is the probabilistic causation of the clinical condition. This evidence is strengthened by the fact that the pathogens identified by our HTS assay were in agreement with the microorganisms that grew by conventional culture methods. Similar to our earlier study, we did detect more than one pathogen in the majority of our endophthalmitis patients, which did not grow in culture, probably due to competition, difference in rate of growth, and/or quorum sensing. The novelty of this study was the high rate of detection of fungal pathogens in 41/57 (71.9%) of our culture-negative samples (Tables S1 and S2), which highlights the geographical location of the patients tested and the at-risk populations which are not being treated with antifungals in the event of a negative microbiological diagnosis. Since fungi are slow growers or are sequestered under the lens/capsular bag [11], detection of these organisms become difficult, resulting in them being reported as culture-negative cases. Additional diagnostic delays might also be attributed by the scarcity of fungal endophthalmitis reported in the literature and the sub-acute nature of early infection, or the difficulty of distinguishing true infection from the occasionally observed sterile inflammation. In our study, a fungal etiology was suspected clinically in only 8/41 culture-negative cases retrospectively. Unfortunately, most outcomes of fungal endophthalmitis are poor as was seen in our study where 20/41 cases diagnosed with fungal endophthalmitis by HTS had an unfavorable outcome (VA > 20/200), likely due to the prolonged time to diagnosis and delay in the administration of appropriate treatment. Another surprising finding in our study was the detection of microbial reads in 4/70 controls tested and though we would not like to rule out the possibility of contamination in these samples, it is also possible that microbial flora in the blood could enter the eye even in non-infectious conditions when there is a break in the blood-retinal barrier. But since these patients were treated on the lines of non-infectious retinal disorders and are doing well without the use of antibiotics, we presume that these controls are not undiagnosed patients. As per institute protocol, it is not a standard practice to perform microbiology cultures on the control samples as clinically they were diagnosed and treated as non-infectious. We also could not process these controls for culture after HTS results were available as the vitreous fluids were exhausted to confirm the HTS findings. Through the counting of sequence reads and calculation of statistical significance, we could recognize that the pathogens detected in the control samples were present in very low reads and our results were otherwise consistently negative for microbial nucleic acids in all test reagents and buffers. A potential drawback of targeted HTS is the detection of microbial contaminants that could be introduced through the reagents and disposables used for processing, or the laboratory environment, which can complicate the analysis and interpretation of results. Therefore, stringent adherence to reagent and workflow quality control procedures is a critical task to maintain a testing environment that is as sterile and nucleic acid–free as possible. The use of negative controls, reagent assessments, and periodic swipe tests are needed to ensure that laboratory and sample cross-contamination are not generating false-positive results. Additionally, the laboratory must be familiar with the commonly encountered ocular flora for the vitreous fluid based on the geographic incidence. The results of this study offer great opportunities for advancing precision medicine in the clinical microbiology laboratory. In the setting of culture-negative endophthalmitis, it validates the clinical judgment by ruling in the disease and helps in administering targeted antimicrobial therapy or ruling out sterile endophthalmitis instead of starting the administration of antibiotics empirically. Additionally, with the implementation of HTS, outbreak isolates may be identified to help and guide infection control efforts and antibiotic stewardship.

A major limitation of our study is that the true relevance of these pathogens in the culture-negative cases could not be validated by real-time PCR or Sanger Sequencing of the amplicons since the samples were exhausted after routine microbiological and HTS analysis, nevertheless we have to understand the clinical setting that each of these samples were clinically suspected and presumed infectious and the probability of detecting a causative pathogen was always high. From the cost-effectiveness per se, targeted HTS may help to avert additional investigations in cases of non-responding infections and therefore, will find utility in diagnostic ocular laboratories throughout the country. To assess the clinical relevance of the identified genera in such conditions, we plan a future prospective clinical trial on samples of complex patient groups of surgical endophthalmitis alone and control groups of patients without clinical suspicion of an infection. Ongoing interactions between clinicians, ocular microbiologists, and bioinformaticians are essential to assess the clinical relevance of these newer technologies in precision medicine. 

## 4. Materials and Methods 

### 4.1. Ethics

This was a prospective study project approved by the L V Prasad Eye Institute-Institutional Review Board (LEC 11-16-112) on 8 November 2016, and the research protocols and study design adhered to the tenets of the Declaration of Helsinki. Informed consent was taken from each patient after recording clinical details prior to study participation.

### 4.2. Clinical Samples

Vitreous fluids from 75 patients diagnosed clinically with presumed infectious endophthalmitis between February 2017 and December 2017 were included in the study and all samples were subjected to routine microbiological evaluation as described earlier [9]. All cases had clinical features suggestive of endophthalmitis including ocular pain, decreased vision, eyelid edema, conjunctival congestion, chemosis, intense AC inflammation, extensive vitreous exudates, corneal infiltrates or a lens abscess, hypopyon, vitritis, and decreased red reflex. They were all clinically diagnosed and treated on lines of endophthalmitis. In our series, we had excluded all cases which had a suspicious diagnosis or where the clinical characteristics were ambiguous.

Briefly, vitreous fluid was collected aseptically from all these cases in the operating room. A standard three port, vitrectomy was done, and a 6 mm infusion cannula was used and the vitreous was aspirated through the vitrectomy probe that was connected to the vitrectomy suction tube. The undiluted (about 0.5 mL) vitreous was collected in a 2 mL disposable syringe attached to the vacuum line of the vitreous cutter and a vacuum was created by mechanical suction. The syringe with the vitreous sample was then capped with a hypodermic needle and transported in a sterile plastic container to the microbiology laboratory of the institute. Following the vitrectomy, in all these 75 patients, an intravitreal injection of antibiotics (vancomycin 1 mg/0.1 mL and amikacin 0.4 mg/0.1 mL or ceftazidime 2.25 mg/0.1 mL) with or without intravitreal dexamethasone (400 µg/0.1 mL) for empirical coverage was administered. Based on clinical suspicion or microbiological evidence of a fungal infection, the patients were given intravitreal amphotericin B (5µg/0.1 mL) additionally along with systemic antifungals. All these patients were also given ciprofloxacin (750 mg twice daily for 6 days) as the systemic antibiotic along with topical ciprofloxacin, atropine, and prednisolone acetate eye drops. 

For the control group, 70 patients undergoing vitrectomy for other retinal disorders (retinal detachment, or macular hole or diabetic retinopathy), that had consented for vitreous tap done during the procedure, during the same period were included. 

### 4.3. PCR Amplification, Illumina HiSeq Sequencing, and NGS Library Preparation

DNA was extracted from the fluids using the QIAamp DNA minikit (Qiagen, Germany), followed by PCR amplification using the V3-V4 hypervariable region of the16S rRNA gene for bacteria [3], and internal transcribed spacer region (ITS2) of the ribosomal small subunit RNA for fungus [4], before purification and sequencing as described earlier [9].

### 4.4. Taxonomic Analysis by NGS

Raw paired-end reads of individual samples were assembled using the FLASH program and filtered to remove low quality reads (average Phred score <30) and chimeric sequences using Prinseq-lite and Usearch61, respectively. The quality reads were then subjected to the open reference operational taxonomic unit (OTU) picking using the QIIME (Quantitative Insights into Microbial Ecology) pipeline [12] with Greengenes (V 13.8) and Unite (V 12.11) OTUs clustered at 97% sequence similarity for bacterial and fungal identification, respectively. Taxonomic classification for denovo-OTUs was made through Wang Classifier with a bootstrap of 80%. Sparse OTUS (<0.001%) were removed from further analysis and taxonomic profiles for both bacterial and fungal data based on OTU annotation were generated at the genera level. Clustered Heatmaps with the rank abundance plot of bacterial and fungal genera were plotted using R software.

### 4.5. Identification of Contaminant Microbial Reads

To avoid any potential contaminant in the reads, we additionally sequenced DNA extraction (blank) controls and removed those taxa (however at a lesser frequency compared to the test groups) from the datasets of all endophthalmitis patients. We reported only those reads from the pathogens that were not present in the negative and blank (water only) control on the same run and library preparation.

## 5. Conclusions

In conclusion, this targeted HTS platform is expected to be potentially game-changing technology in clinical microbiology, especially in culture-negative infections, while also broadening the repertoire of tools readily in-hand for health decision making in the future.

## Figures and Tables

**Table 1:** Clinical and microbiological details of the study patients diagnosed clinically as infectious endophthalmitis.

	Culture Positive (18)	Culture Negative (42)
**Demographic Characteristics**		
Age in years (mean:range)	37.7:2–74	38.7:2–81
Sex (male:female)	15:3	28:14
**Diagnosis**		
Traumatic	8	24
Post-operative	6	15
Endogenous	4	3
**Initial Visual Acuity**		
Eviseration/Phthisis	0	0
<(20/200)	17	34
>(20/20)–(20/200)<	1	8
=20/20	0	0
**Microbiology**	*Streptococcus pneumonia* (4) *Streptococcus mitis* (1)	
**Bacteria**	*Staphylococcus epidermidis* (4) *Staphylococcus haemolyticus* (1) *Enterococcus casseliflavus* (1) *Enterococcus faecalis* (1) *Klebsiella pneumoniae* (1) *Corneybacterium pseudodiphtherticum* (1) *Bacillus licheniformis* (1)	
**Fungi**	*Candida trophicalis* (1) *Fusarium solani* (1) *Candida albicans* (1)	

**Table 2:** High-throughput sequencing (HTS) reads and taxonomic classification of bacterial culture-positive samples.

S. No.	Sample ID	Culture Report	HTS Identification Taxonomic Lineage with Abundance
1	E1	*Streptococcus pneumoniae*	*Streptococcus =* 99%
2	E2	*Enterococcus casseliflavus*	Enterococcus = 85% Unclassified = 10%
3	E3	*Staphylococcus epidermidis*	*Staphylococcus* = 89% Unclassified = 10%
4	E4	*Streptococcus pneumoniae*	*Streptococcus* = 97%
5	E5	*Klebsiella pneumoniae*	*Klebsiella =* 90% *Erwinia =* 5%
6	E6	*Staphylococcus epidermidis*	*Staphylococcus =* 92% *Planococcaceae =* 6%
**7**	**E7**	***Corneybacterium pseudodiphtherticum***	***Pseudomonas =* 94%** ***Pseudomonadaceae =* 5%**
8	E8	*Streptococcus pneumoniae*	*Streptococcus* = 94%
9	E9	*Staphylococcus epidermidis*	*Staphylococcus =* 86% *Planococcaceae =* 7% Unclassified = 3%
10	E10	*Enterococcus faecalis*	*Enterococcus =* 85% Unclassified = 10% Enterococcaceae = 4%
11	E11	*Streptococcus mitis*	*Streptococcus =* 95%
12	E12	*Staphylococcus epidermidis*	*Pseudomonas =* 65% *Methylobacterium =* 14% *Staphylococcus =* 13%
13	E13	*Bacillus licheniformis*	*Bacillus =* 86% *Staphylococcus =* 3%
14	E14	*Staphylococcus haemolyticus*	*Methylobacterium =* 84% *Staphylococcus =* 4% *Morganllea =* 4%
15	E15	*Streptococcus pneumoniae*	*Streptococcus =* 94%

**Table 3:** HTS reads and taxonomic identification in fungal culture-positive samples.

S. No.	Sample ID	Culture Report	HTS Identification Taxonomic Lineage with Abundance
1	E27	*Candida trophicalis*	*Candida =* 99%
2	E28	*Fusarium solani*	*Fusarium* = 59% Unclassified = 41%
3	E29	*(1) Candida albicans* *(2) Staphylococcus aureus*	*Candida* = 100%

**Table 4:** HTS reads and taxonomic lineage identified in control samples.

S. No.	Sample ID	HTS Identification Taxonomic Lineage with Abundance
1	E17C	Enterobacteriaceae = 53%, *Paracoccus* = 13%, *Acinetobacter* = 5%, *Staphylococcus* = 4% *Enhydrobacter* = 3%*, Corynebacterium* = 3% Rhodobacteraceae = 3%
2	E26C	*Streptococcus* = 78%*, Propionibacterium* = 7% *Abiotrophia* = 3%
3	E53C	*Enhydrobacter* = 32%*, Sphingobium* = 21% *Flavobacterium* = 11%*, Pseudomonas* = 9% Caulobacteraceae = 3%
4	E54C	Enterobacteriaceae = 52%, *Corynebacterium* = 9% *Citrobacter* = 8%, *Lactobacillus* = 5% *Finegoldia* = 4%, *Erwinia* = 3%

## Supplementary Materials

Supplementary materials can be found at https://www.mdpi.com/2076-2607/7/10/411/s1. Table S1. Reads assigned to bacterial OTUs for 26 bacterial microbiome libraries. Sparse OTUs (with <0.001% reads) were removed. Table S2. Reads assigned to fungal OTUs for 39 fungal microbiome libraries. Sparse OTUs (with <0.001% reads) were removed.
